# Supplementary figures and images for: Efficacy of epetraborole against Mycobacterium abscessus is increased with norvaline
Source: PLoS Pathog. 2021 Oct 12;17(10):e1009965. doi: 10.1371/journal.ppat.1009965 (PMC8535176; doi:10.1371/journal.ppat.1009965)

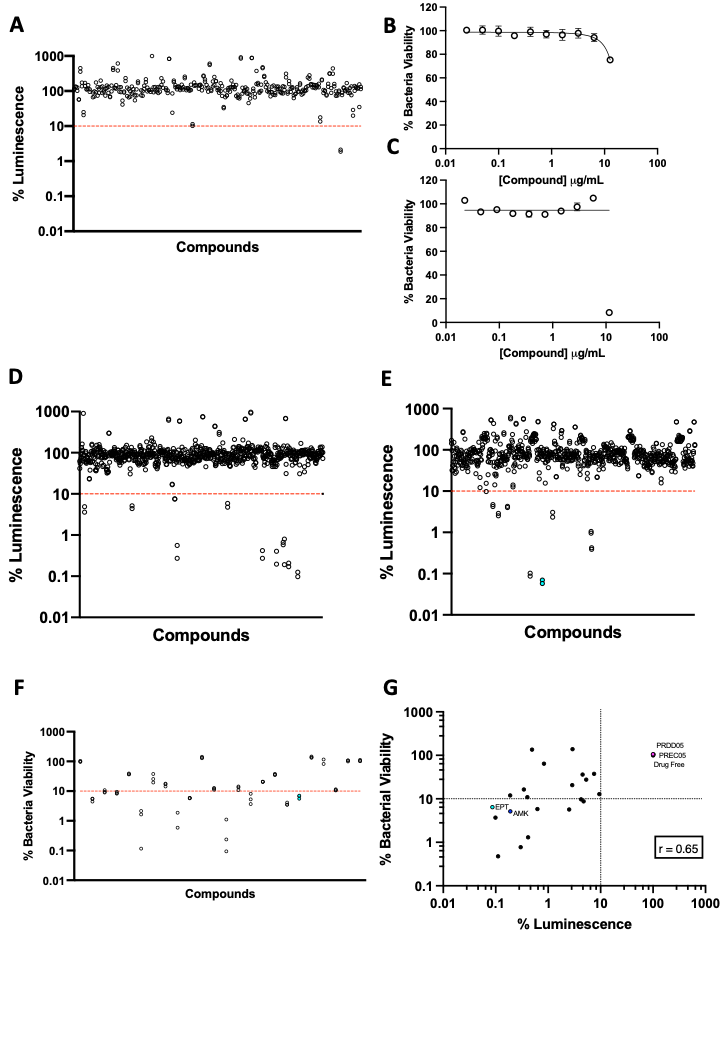

Supplement: S1 Fig — M. abscessus phenotypic screening with 176 TB-active compounds from GSK (A-C) and MMV (D-F). A Luminescence relative to drug free conditions using M. abscessus lux. Data is shown in duplicate. B-C Secondary screening of primary hits using REMA on M. abscessus ATCC 19977. % bacteria viability relative to drug free conditions. Data is shown as mean ± SD from technical triplicates. Dashed red line indicates 10% viability threshold when screened at 10μM. Three hits were identified from primary screen however, all three did not pass the secondary screen. No active compounds were identified from this library. D MMV Pathogen box and E Pandemic response box; luminescence relative to drug free conditions using M. abscessus lux. Data is shown in duplicate. F Secondary screening of primary hits using REMA on M. abscessus ATCC 19977. % bacteria viability relative to drug free conditions. Data is shown in technical triplicate. Dashed red line indicates 10% viability threshold when screened at 10μM. G Correlation between hits from luminescence primary screen and bacterial viability secondary screen. Correlation calculated using non-parametric Spearman correlation. 20 compounds passed the primary screen and 9 passed the secondary screen. Only three compounds were still active after acquiring fresh batch and displayed dose-dependent activity (3/800). EPT in cyan. AMK in blue as positive control. Two negative hits from luminescence screen in magenta used as negative controls. Drug free in black used as media control. (TIFF) [file ppat.1009965.s001.tiff]

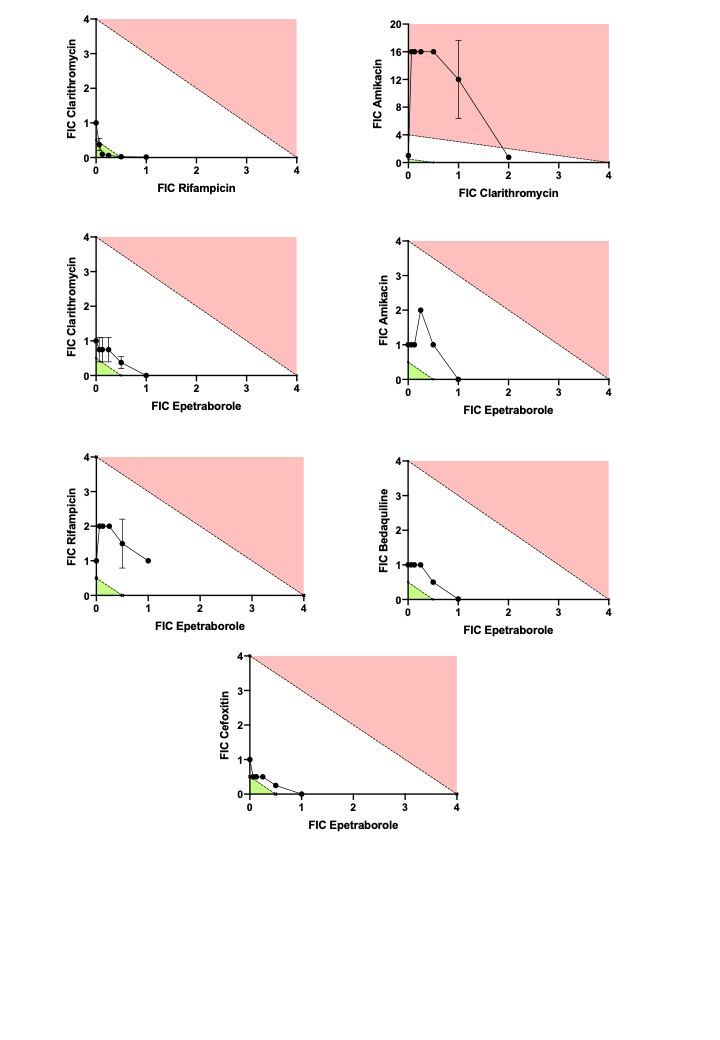

Supplement: S2 Fig — Green area indicates synergy (FICI < 0.5); red area indicates antagonism (FICI ≥ 4.0); white area indicates indifferent. RIF and CLR, and AMK and CLR are used as synergy and antagonism controls, respectively. Data shown is from checkerboard assays done in technical duplicate. (TIFF) [file ppat.1009965.s002.tiff]

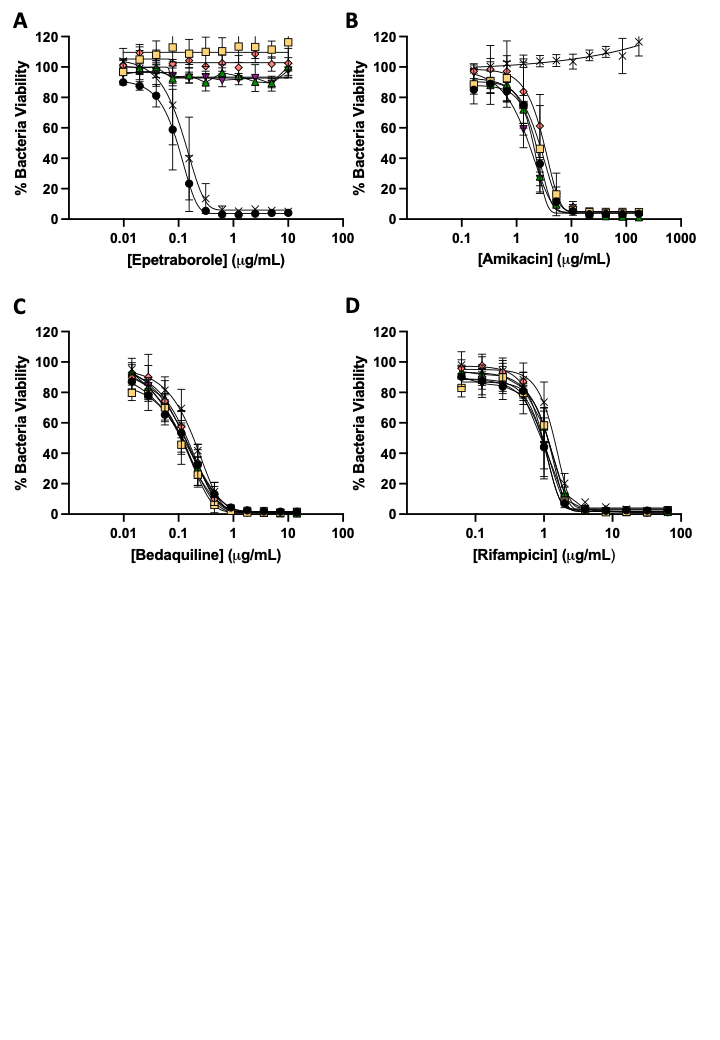

Supplement: S3 Fig — A-D Dose-response curves of isolated EPT (10X, 20X, 40X MIC) mutants or AMK (40X MIC) mutant. AMK was used as control compound for mutant isolation. RIF and BDQ were used for cross-resistance verification. ATCC 19977 (black circles), D436H mutant-1 (yellow squares), D436H mutant-2 (pink diamonds), D436H mutant-3 (green triangles), D436H mutant-4 (purple inverted triangles), AMK 40X (crosses). Data is mean ± SD from two independent experiments. (TIFF) [file ppat.1009965.s003.tiff]

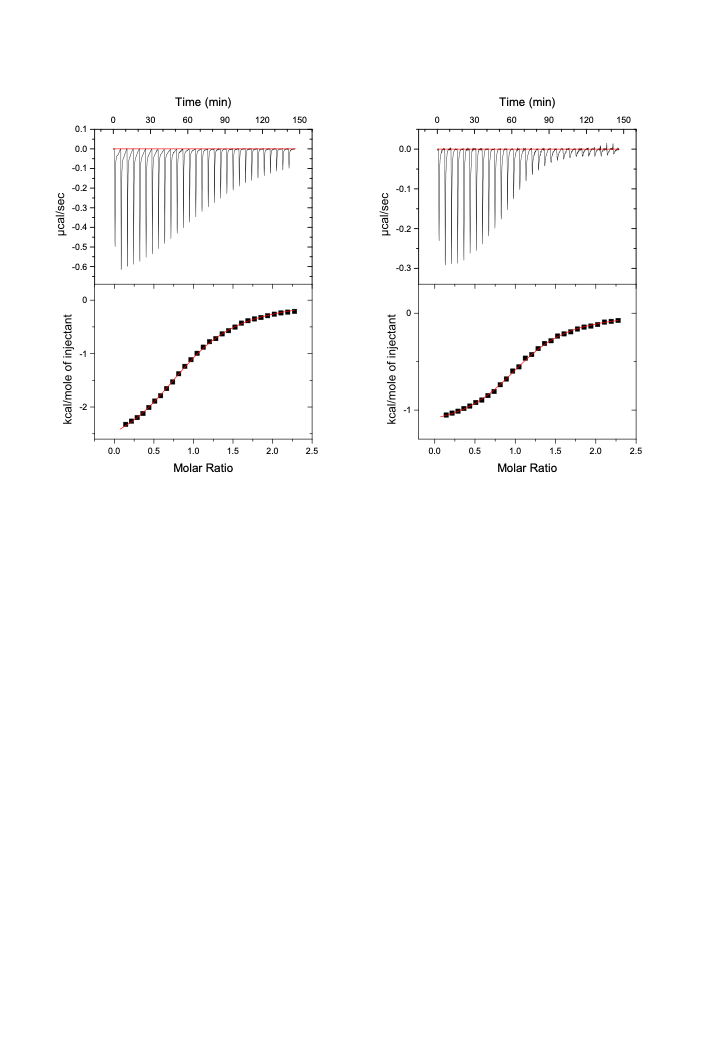

Supplement: S4 Fig — Heat of injection (upper panel) and single-site binding model of the integrated isotherm (lower panel). Mabs LeuRS (left) or Mtb LeuRS (right) editing domains bound to EPT. (TIFF) [file ppat.1009965.s004.tiff]

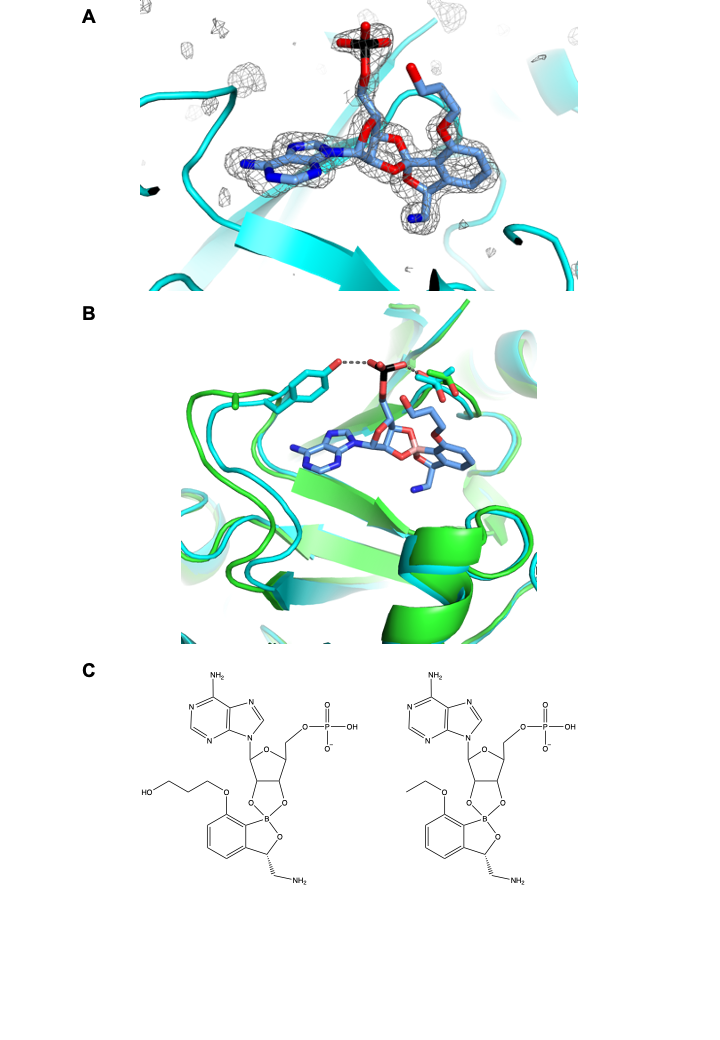

Supplement: S5 Fig — A Difference maps for the EPT-AMP adduct in 7N12. Unbiased FO-FC difference maps (2.8σ), calculated with phases from a model that never included ligand. B Comparison of apo (green, PDB 7N11) and co-complex (cyan, PDB 7N12) structures of M. abscessus LeuRS bound to EPT-AMP. C Benzoxaborole inhibitors of LeuRS. (Left) EPT-AMP adduct bound to M. abscessus LeuRS. (Right) BNZ-AMP adduct bound to M. tuberculosis LeuRS. (TIFF) [file ppat.1009965.s005.tiff]
