# Supplementary material for: Efficacy of epetraborole against Mycobacterium abscessus is increased with norvaline
Source: PLoS Pathog. 2021 Oct 12;17(10):e1009965. doi: 10.1371/journal.ppat.1009965 (PMC8535176; doi:10.1371/journal.ppat.1009965)
Supplement: S1 Table — G, Glycerol; A, Acetate; Tw80, Tween-80; CaMH, Cation-adjusted Muller-Hinton. (DOCX) [file ppat.1009965.s006.docx]

|  | **Media** | | | |
| --- | --- | --- | --- | --- |
|  | **7H9 G + Tw80** | **7H9 G - Tw80** | **7H9 A + Tw80** | **CaMH + Tw80** |
| MIC_90_ (µg/mL) | 0.06 $\pm$ 0.02 | 0.011 $\pm$ 0.008 | 0.016 $\pm$ 0.005 | 0.5 $\pm$ 0.2 |

G, Glycerol; A, Acetate; Tw80, Tween-80; CaMH, Cation-adjusted Muller-Hinton.
