# Supplementary material for: Efficacy of epetraborole against Mycobacterium abscessus is increased with norvaline
Source: PLoS Pathog. 2021 Oct 12;17(10):e1009965. doi: 10.1371/journal.ppat.1009965 (PMC8535176; doi:10.1371/journal.ppat.1009965)
Supplement: S2 Table — aMorphology as determined by smooth (S) or rough (R) colonies on 7H10 agar. Drugs used: EPT, Epetraborole; AMK, Amikacin; RIF, Rifampicin; BDQ, Bedaquiline; CFX, Cefoxitin; CLR, Clarithromycin. (DOCX) [file ppat.1009965.s007.docx]

|  | | | **MIC (µg/mL)** | | | | | |
| --- | --- | --- | --- | --- | --- | --- | --- | --- |
| **Isolate** | **Subspecies** | **Morphology^a^** | **EPT** | **AMK** | **RIF** | **BDQ** | **CFX** | **CLR** |
| ATCC 19977 | *abscessus* | S | 0.063 | 4.8 | 9.1 | 0.67 | 12 | 0.90 |
| ATCC 19977 | *abscessus* | R | 0.11 | 2.4 | 1.8 | 0.27 | 12 | 0.65 |
| MT16_1065 | *abscessus* | S | 0.041 | 2.1 | >24 | 0.36 | 13 | 3.4 |
| MT16_6490 | *abscessus* | S | 0.030 | 5.7 | >24 | 0.83 | 13 | 2.9 |
| MT15_1748 | *massiliense* | R | 0.022 | 14 | 9.1 | 0.23 | 26 | 0.068 |
| MT15_1749 | *massiliense* | R | 0.014 | 8.8 | 6.7 | 0.40 | 16 | 0.082 |
| Paris 167 | *bolletii* | R | 0.027 | 4.8 | 7.4 | 0.39 | 16 | 2.8 |
| AV | *bolletii* | S | 0.046 | 7.0 | >24 | 1.3 | 15 | 0.15 |

^a^Morphology as determined by smooth (S) or rough (R) colonies on 7H10 agar. Drugs used: EPT, Epetraborole; AMK, Amikacin; RIF, Rifampicin; BDQ, Bedaquiline; CFX, Cefoxitin; CLR, Clarithromycin
