# Supplementary material for: Efficacy of epetraborole against Mycobacterium abscessus is increased with norvaline
Source: PLoS Pathog. 2021 Oct 12;17(10):e1009965. doi: 10.1371/journal.ppat.1009965 (PMC8535176; doi:10.1371/journal.ppat.1009965)
Supplement: S3 Table — aAcid fast (AF); gram positive (+); gram negative (-). EPT, Epetraborole; AMK, Amikacin; RIF, Rifampicin; ERV, BDQ, Bedaquiline; CFX, Cefoxitin; CLR, Clarithromycin. Species: Mycobacterium abscessus, Mycobacterium avium hominissuis, Mycobacterium avium intracellulaire, Mycobacterium tuberculosis H37Rv, Mycobacterium tuberculosis Erdman, Bacillus cereus, Corynebacterium glutamicum, Escherichia coli, Pseudomonas aeruginosa. (DOCX) [file ppat.1009965.s008.docx]

|  | | **MIC (µg/mL)** | | | | | |
| --- | --- | --- | --- | --- | --- | --- | --- |
| **Species** | **Gram^a^** | **EPT** | **AMK** | **RIF** | **BDQ** | **CFX** | **CLR** |
| *M. abscessus* | AF | 0.063 | 4.8 | 9.1 | 0.67 | 12 | 0.90 |
| *M. avium hominissuis* | AF | 2.7 | 5.6 | 0.063 | 0.038 | 4.7 | 0.18 |
| *M. avium intracellulaire* | AF | 2.7 | 3.8 | 0.063 | 0.014 | 0.42 | 0.014 |
| *M. tuberculosis H37Rv* | AF | 0.46 | 0.30 | 0.0063 | 0.26 | >98 | >1.3 |
| *M. tuberculosis Erdman* | AF | 0.19 | 0.48 | 0.0065 | 0.061 | >98 | >1.3 |
| *B. cereus* | + | >2.7 | 64 | <0.063 | >7.8 | >98 | >8.2 |
| *C. glutamicum* | + | 2.0 | 0.064 | <0.063 | >7.8 | 98 | >8.2 |
| *E. coli* | - | 5.7 | 0.15 | 3.9 | >7.8 | 4.0 | >8.2 |
| *P. aeruginosa* | - | >2.7 | 0.22 | 15 | >7.8 | >98 | >8.2 |

^a^Acid fast (AF); gram positive (+); gram negative (-). EPT, Epetraborole; AMK, Amikacin; RIF, Rifampicin; ERV, BDQ, Bedaquiline; CFX, Cefoxitin; CLR, Clarithromycin. Species: *Mycobacterium abscessus*, *Mycobacterium avium hominissuis*, *Mycobacterium avium intracellulaire*, *Mycobacterium tuberculosis* H37Rv, *Mycobacterium tuberculosis* Erdman, *Bacillus cereus*, *Corynebacterium glutamicum*, *Escherichia coli*, *Pseudomonas aeruginosa*
