# Supplementary material for: Efficacy of epetraborole against Mycobacterium abscessus is increased with norvaline
Source: PLoS Pathog. 2021 Oct 12;17(10):e1009965. doi: 10.1371/journal.ppat.1009965 (PMC8535176; doi:10.1371/journal.ppat.1009965)
Supplement: S4 Table — aN.D: not determined. bMIC90 values on 7H10 agar used in this experiment were 4.0 μg/mL and 0.27 μg/mL for amikacin and epetraborole, respectively. (DOCX) [file ppat.1009965.s009.docx]

|  | **Resistance frequency^a^** | | |
| --- | --- | --- | --- |
| **Compound^b^** | **10X** | **20X** | **40X** |
| Amikacin | 1.3 X 10^-8^ | N.D | N.D |
| Epetraborole | 2 X 10^-9^ | 1 X 10^-9^ | 2 X 10^-9^ |

^a^N.D: not determined. ^b^MIC_90_ values on 7H10 agar used in this experiment were 4.0 µg/mL and 0.27 µg/mL for amikacin and epetraborole, respectively.
