# Supplementary material for: Efficacy of epetraborole against Mycobacterium abscessus is increased with norvaline
Source: PLoS Pathog. 2021 Oct 12;17(10):e1009965. doi: 10.1371/journal.ppat.1009965 (PMC8535176; doi:10.1371/journal.ppat.1009965)
Supplement: S5 Table — (DOCX) [file ppat.1009965.s010.docx]

| **Primers** | **Function** | **Sequence** | **Source** |
| --- | --- | --- | --- |
| 1.  pMV306hsp60_leuS_F | Cloning | GATGATCTGCAGAACCGAAACCCAGCACGACG | This study |
| 2.  pMV306hsp60_leuS_R | Cloning | GATGATAAGCTTCTAGACGACCAGGTTCACCATG | This study |
| 3. pMV306hsp60_leuS_F1 | Sequencing | GTAAGTAGCGGGGTTGCCGT | This study |
| 4. pMV306hsp60_leuS_R1 | Sequencing | GCTGCCGCTTGTAGTTGACG | This study |
| 5. pMV306hsp60_leuS_F2 | Sequencing | TGCAGACCGGCACCCATCC | This study |
| 6. pMV306hsp60_leuS_R2 | Sequencing | TTTGACCTTGTCCGGCCAGT | This study |
| 7. pMV306hsp60_leuS_F3 | Sequencing | CGCCTACTCCGACAGGTTGA | This study |
| 8. pMV306hsp60_leuS_R3 | Sequencing | CCGGCAAACCGAAGGTGTT | This study |
| 9. pMV306hsp60_leuS_F4 | Sequencing | ATGGCACCGGTGCCATCAT | This study |
| 10. pMV306hsp60_leuS_R4 | Sequencing | GCGGCATCACGTTGGTGTC | This study |
| 11. pMV306hsp60_leuS_F5 | Sequencing | AATGTCGAGCTGGACCTCGG | This study |
| 12. pMV306hsp60_leuS_R6 | Sequencing | GCAGCGTATCGGCACCATAGT | This study |
| 13. pMV306hsp60_leuS_F6 | Sequencing | GCCTCAAGAACTCGATCTCGC | This study |
| 14. pMV306hsp60_leuS_R6 | Sequencing | TGGCAGTCGATCGTACGCTAG | This study |
| 15. CRISPRi_leuS_sgRNA_F | CRISPRi | GGGAACTCTTGTTGCACCTCTGCGCCCTG | This study |
| 16. CRISPRi_leuS_sgRNA_R | CRISPRi | AAACCAGGGCGCAGAGGTGCAACAAGAGT | This study |
