# Supplementary material for: Efficacy of epetraborole against Mycobacterium abscessus is increased with norvaline
Source: PLoS Pathog. 2021 Oct 12;17(10):e1009965. doi: 10.1371/journal.ppat.1009965 (PMC8535176; doi:10.1371/journal.ppat.1009965)
Supplement: S6 Table — (DOCX) [file ppat.1009965.s011.docx]

| **Gene** | **Function** | **Variant** | |
| --- | --- | --- | --- |
|  |  | **20X** | **40X** |
| **38bp upstream MAB_2723c** | **3-oxoacyl-[ACP] reductase** | **del CCAAGGAACCGCA** |  |
| **MAB_4932c** | **Leucyl-tRNA synthetase** | **T1261G**  **(Y421D)** | **G1306C**  **(D436H)** |
| **MAB_3268c** | **Conserved hypothetical protein** | **A266G**  **(Q89R)** |  |
| **MAB_4735** | **Putative starvation-induced DNA protecting protein** | **G230A**  **(R77H)** |  |
| **MAB_4814** | **Conserved hypothetical protein** | **A3576G**  **(E1192E)** |  |
