# Supplementary material for: Efficacy of epetraborole against Mycobacterium abscessus is increased with norvaline
Source: PLoS Pathog. 2021 Oct 12;17(10):e1009965. doi: 10.1371/journal.ppat.1009965 (PMC8535176; doi:10.1371/journal.ppat.1009965)
Supplement: S7 Table — (DOCX) [file ppat.1009965.s012.docx]

|  | *M. abscessus*  LeuRS editing domain | *M. abscessus*  LeuRS editing domain in complex with epetraborole-AMP adduct |
| --- | --- | --- |
| **Data collection** |  |  |
| Space group | P 2_1_ 2_1_ 2_1_ | C 1 2 1 |
| Cell dimensions |  |  |
| *a*, *b*, *c* (Å) | 37.25, 51.59 116.15 | 113.439, 37.1074, 100.287 |
| α, β, γ (°) | 90, 90, 90 | 90, 112.279, 90 |
| Resolution (Å) | 1.69-50.00 (1.69-1.72) * | 1.52-92.97 (1.52-1.55) * |
| R_sym_ | 0.263 (0.824) * | 0.100 (1.071) * |
| *I* / σ*I* | 4.4 (0.5) * | 7.7 (0.3) * |
| Completeness (%) | 76.8 (5.5) * | 94.21 (49.23) * |
| Redundancy | 6.8 (1.1) * | 5.54 (3.08) * |
|  |  |  |
| **Refinement** |  |  |
| Resolution (Å) | 2.1 | 1.7 |
| No. reflections | 14741 | 109484 |
| *R*_work_ / *R*_free_ | 0.1841 / 0.2258 | 0.1843 / 0.2265 |
| No. atoms | 2968 | 5964 |
| Protein | 2822 | 5435 |
| Ligand  Ion | /  15 | 126  5 |
| Water | 131 | 384 |
| *B*-factors |  |  |
| Protein | 25.44 | 20.14 |
| Ligand  Ion | /  63.20 | 19.98  56.13 |
| Water | 31.17 | 31.70 |
| R.m.s. deviations |  |  |
| Bond lengths (Å) | 0.008 | 0.013 |
| Bond angles (°) | 0.999 | 1.216 |
|  |  |  |
| **PDB accession code** | 7N11 | 7N12 |
