# Supplementary material for: Efficacy of epetraborole against Mycobacterium abscessus is increased with norvaline
Source: PLoS Pathog. 2021 Oct 12;17(10):e1009965. doi: 10.1371/journal.ppat.1009965 (PMC8535176; doi:10.1371/journal.ppat.1009965)
Supplement: Attachment — Submitted filename: Revewier comments with answers and edits.docx [file ppat.1009965.s013.docx]

Reviewer comment in grey. Our response in black. Text change in red.

**Part I - Summary**
Please use this section to discuss strengths/weaknesses of study, novelty/significance, general execution and scholarship.

Reviewer #1: The manuscript entitled "Efficacy of epetraborole against Mycobacterium abscessus is increased with norvaline" by Sullivan, describes the activity of epetraborate and norvaline against M. abscessus. Overall the paper is very interesting and generally well supported by experimental results. I think this paper could strongly benefit from enzymatic data. Structures look very good and support the results of the paper. There are several issues that should be addressed before the paper can be accepted.

Thank you for the positive overall feedback and the specific suggestions which are addressed in turn below.

1. The beginning of the abstract needs to be rewritten. The first few sentences are short and seem disjointed. It reads more like bullet points.

The abstract was modified with smoother transition.

2. Line 103 – The authors used two different methods to measure viability in their whole cell active compound screen, namely luminescence and resazurin. I think it would be helpful to show the correlation between these two screens. I do not believe it changes validity of results, but it would be good to know how many compounds were active on luminescence, resazurin or both.

An additional panel (g) was added to Supplementary Figure 1 with visual representation of correlation between hits from primary and secondary screens. Text has been modified to include the 20 hits from luminescence primary screen and the 9 hits from resazurin secondary screen. These numbers were also included in the caption of Supplementary Figure 1.

“Primary hits (20 compounds) were tested in a secondary screen using the resazurin microtiter assay (REMA) at 10 µM on the *M. abscessus* ATCC 19977 strain. Secondary hits (9 compounds) were then tested in a dose-response assay on *M. abscessus* ATCC 19977 from a fresh solid compound to determine MIC_90_.”

3. Line 114 – The author claims that EPT lost potency in cation-adjusted Muller Hinton. This seems like an important finding. I believe the authors would make a stronger statement if they could at least provide a at least some speculation on mode of action of EPT.

Text has been modified to include possible mechanism for loss of activity in Mueller-Hinton broth. Mueller-Hinton has been shown previously to affect antimycobacterial MICs (see Aziz *et al*. 2017. *Antimicrob Agents Chemother*. 61:e00155-17). It is speculated that divalent cations chelate antimicrobials and limit their uptake. (see Fass, R.J., Barnishan, J. 1979. *Antimicrob Agents Chemother*. 16,4:434-438)

“Lower activity of EPT in CaMH media is unsurprising as it was previously shown that rifamycins [18] as well as other antimicrobials [19] lose activity in CaMH media (0.5mg/L Ca^2+^ and 0.05mg/L Mg^2+^ in 7H9; 20-25mg/L Ca^2+^ and 10-12.5mg/L Mg^2+^ in CaMH). It is thought that some antimicrobials may chelate divalent metal ions, thus limiting their uptake into cells.”

4. Line 144 – The author reference fig 1C. I found this figure to be challenging to navigate, due to the number of experiments being shown in each panel (MIC of EPT and CLR, with incubation on EPT or CLR, with and without Passage for 6 days). I found myself constantly having to go back to the legend when trying to compare datasets. I think it may be beneficial to split this figure in more panels to make it easier to understand

Figure 1C has been split into 4 quadrants based on incubation time and the antibiotic under investigation.

5. Fig 1C – on the 14 day figure, it seems like pre exposure to EPT, sensitized cells to both EPT and CLR. Especially when comparing the with and without 6 days of passage. The fold change is not large, but the small error bars for these data sets suggest it is statistically significant. Biological significance is however debatable.

The white bars (drug free control) are 6 data points pooled. 3 data points from initial MIC followed by 3 data points from 6-day passaged MIC. The fold change of each MIC was in relation to the 6 pooled data points. We thank the reviewer for their concern and reanalyzed the data. The previous figure did not use the appropriate comparisons to show induction. Each of the initial MIC readings is now compared to the average of all 3 initial readings; each of the passaged MIC measurements is now compared to the average of all 3 passaged measurements. Pooling the data produced a bimodal distribution in the drug free control due to the prolonged passaging of the cultures. (3 data points from day 1 of experiment cluster together while the 3 data points from day 20 of experiment cluster together) From these data, the groups of interest by the reviewer are still statistically insignificant and would also be biological insignificant as the change in MIC is 2-fold. This would be error in serial 2-fold dilutions in the assay over replicates.

6. Line 154 – The authors found no synergy between EPT and other antimycobacterial drugs, yet the text reads “indicating the potential for a future multidrug regime”. I understand they are mostly pointing out that EPT is at least not antagonistic, but I don’t know if these results demonstrate strong potential for combination therapy.

The wording has been changed to emphasize lack of antagonism with no mention of strong potential for combination therapy and left it as an open question to whether EPT could be useful in multidrug setting.

“To ensure EPT would not hamper a multidrug regimen, we performed checkerboard assays with common antimycobacterial drugs. In the checkerboard assays, we used RIF/CLR and CLR/AMK as the synergy and antagonism controls, respectively [24,25]. We did not observe antagonism between EPT and various antimycobacterial agents that target a range of cellular processes (S2 Fig). Whether EPT should be included in a multidrug regimen remains to be determined.”

7. Line 161 – Wrong figure name. I believe this should be Fig 2A not 1A

Thank you. Correction has been made.

8. Line 167 – The authors claim that there was only minimal benefit at 10ug/mL, but significantly improved at 40ug/mL. However, at day 11, the probability of survival is the same in both dosages. Significantly higher than untreated. Please explain.

Figure 2d clearly shows a delay in larval killing when a 40 µg/ml EPT treatment was applied as compared to a 10 µg/ml treatment, which is correlated with a more intense reduction in the bacterial burden at 4 and 6 dpi, as illustrated in Figure 2f. However, since drug treatments are usually maintained for only 5 days (Bernut *et al*. 2014. *Antimicrob. Agents Chemother*. 58:4054-63), as indicated by a red bar in Figure 2d, this may explain why that at later stages (up to 11 days post-infection) the 10ug/mL and 40ug/mL groups converge when EPT is no longer replenished.

“The protection provided by EPT is maintained throughout the 5-day treatment course and was correlated with decreased bacterial burdens beginning 4 dpi demonstrated by fluorescent pixel counting (Fig 2c and Fig 2d).”

9. Fig 2B – I believe this experiment should have been carried out longer. Although the 10ug trace started off closely matching the untreated, they separate in the later days. The authors repeatedly mention that abscessus is notoriously difficult to treat, with regimen taking 18 months, yet stopped recording data 11 days post infection. Zebrafish can live for 3 years. Furthermore at 11 days, survivability was still 40% in the untreated cohort, and was not showing sign of plateauing.

Under non-experimental conditions, zebrafish can live for 3 to 4 years. However, this is not possible under our experimental settings. All experiments here were carried out in zebrafish larvae, characterized by the sole innate immune response, while adults possess both the innate and adaptive immune systems. Typically, mycobacterial infections in larvae last for 10-12 days (see Takaki *et al*. 2013. *Nat. Protoc*. 8: 1114-1124) after which they have completely absorbed their yolk-sac nutrients as their sole nutrient source. In addition, testing the *in vivo* efficacy of drug against *M. abscessus* in adult fish has not been reported yet in the literature. Although of interest, this warrants future methodological developments and ethical approval.

10. Line 185 – The authors conclude that the mechanism of resistance must be specific to EPT. However, they only test 3 other drugs. Although it is true that this seems EPT dependent, testing 3 other compounds, with unrelated mechanisms of action, is in my opinion not enough to say it is EPT specific.

Specificity of mechanism of resistance has been removed.

“In order to confirm that isolated resistant mutants were true mutants to EPT, we performed REMA using a panel of antimycobacterial agents on the four mutants (one selected on 10X, one on 20X, and two on 40X MIC_90_ of EPT). We also screened one AMK mutant. As illustrated in S3 Fig, the four EPT mutants were resistant up to 10 µM EPT, while maintaining susceptibility to AMK, bedaquiline (BDQ), and RIF. Likewise, the AMK control mutant was only resistant to AMK.”

11. Line 186 – The authors decided to use a focused approach to identify the mutation in leuS based on literature. However, with only 4 four mutants, I think doing whole genome sequence would have been feasible. It is entirely possible that each mutant had to accumulate more than the D436H mutation. Since the resistance frequency is higher than expected, this is a possibility.

Whole-genome sequencing has now been done and the new data are included. Compared to the reference strain, the 20X mutant accumulated 4 additional variants in addition to its *leuS* SNP. The 40X mutant however, contained a single variant, the SNP in *leuS* previously identified by Sanger sequencing.

“This was further supported with whole-genome sequencing of the *M. abscessus* ATCC 19977 reference strain, one mutant isolated at 20X MIC, and one mutant isolated at 40X MIC. When compared to the reference strain, five variants were identified in the 20X mutant including a T to G transition at position 1261 in leuS which resulted in a Y421D substitution. Furthermore, the G to C transversion at position 1306 which lead to the D436H substitution was confirmed in the 40X mutant (S6 Table).”

12. Fig 3c – WT cells can become resistant by transforming with a mutant leuS, indicating the enzyme plays a role in the mechanism of resistance. However, the level of resistance is intermediate. I think this should be addressed in the paper to offer some hypotheses. Relative expression of WT leuS vs Mutant leuS in this strain could also explain the results. It is also possible the resistant mutant strain has other mutation contributing to the resistance.

From the WGS data added to the manuscript, the 40X mutant only required the D436H substitution to become resistant to epetraborole. Indeed, the leuS gene with the G1306C mutation required for D436H substitution only provided partial resistance to epetraborole in a wild type background. However, the vector used was the integrative vector pMV306 and the mutant gene was under control of the *hsp60* promoter from *M. bovis*. One explanation for this partial phenotype is that the *hsp60* promoter is integrated and is not expressed as easily compared to the episomal vector pMV206 with *hsp60* promoter. A second explanation is that the *hsp60* promoter from *M. bovis* is considered constitutive however, *M. abscessus* may not use this promoter constitutively. A third explanation is that the *hsp60* promoter may not express the mutant *leuS* to same levels as the *leuS* promoter.

“Only EPT sensitive *M. abscessus* complemented with the mutant *leuS* (pMVhsp60_leuS^D436H^) and not the wildtype *leuS* (pMVhsp60_leuS^D^) had increased resistance to EPT (Fig 3c). The integrative complement system using pMVhsp60 may not have provided a complete phenocopy of the true mutant when compared to the episomal equivalent. Also, *M. abscessus* may not use the *hsp60* promoter from *M. bovis* with optimal efficiency. Lastly, the *hsp60* promoter may not operate with the same strength as the *leuS* promoter. “

13. Line 215 – The authors hypothesize that EPT is more potent against M. abscessus than M. tuberculosis because it has higher affinity to LeuRS active site in M. abscessus. Binding data does not strongly support that this is the case. However, in order to demonstrate stronger inhibition of the M. abscessus enzyme, I believe an enzymatic assay would be very important. The difference in whole cell potency between species could easily be due to difference in cell permeability, efflux pump or many other cellular processes that can affect potency of drugs. Aminoacyl-tRNA synthetase can be easily assayed by measuring released pyrophosphate or AMP.

The text has been modified to emphasize that EPT is more potent against M. abscessus than M. tuberculosis on the whole-cell level, which could be the result of various factors. Indeed, the ITC binding data did not support our hypothesis. We attempted to express and purify the full-length LeuRS however, it was produced in the insoluble fraction in E. coli under various conditions assayed. In the end, this is left as an open question to be pursued in the future.

“Other possibilities explaining the difference in whole-cell activity may be linked to differential expression level of *leuS* in both species, the lack of efflux pumps in *M. abscessus*, or the inability of *M. abscessus* to survive conditions that mimic leucine starvation, although these remain to be addressed.”

14. PDB- 7N12 I could not access the structure however, the I/sigma is, I believe, too low in the PDB validation document at 0.2. I believe resolution should be cut.

The PDB validation program chose the threshold of 1.52 A based on a CC_1/2_ > 0.3. Using CC_1/2_ instead of I/sigma(I) as the criterion to claim the resolution of structures has become a new standard in recent years because it has been shown that weak data is still useful. However, our claim was more conservative. In contrast to the validation program, we chose the criterion of I/sigma(I) = 1 to cut the resolution of the structure at 1.7 A. The structure will be released upon publication.

15. Line 252 – Their final conclusion based on structure data seem to contradict their hypothesis that EPT has higher affinity for the M. abscessus enzyme. If this is the case, I believe authors should reflect back on the hypothesis of line 215.

The text has been modified to emphasize better whole-cell activity against M. abscessus than M. tuberculosis even with similar binding affinities/binding pockets. We have left this as an unanswered question.

“the MIC_90_ for *M. abscessus* whole-cell activity is 7-fold lower (MIC_90Mabs_ 0.063 µg/mL vs MIC_90Mtb_ 0.46 µg/mL).”…“From the structural data, we propose that EPT binds LeuRS from *M. abscessus* and *M. tuberculosis* in a similar manner with similar affinity, providing a shared pathway for future structure-activity relationship analysis.”

16. Line 317 – Using Norvaline in order to reduce resistance is, in my opinion, very clever. However, I believe that in order to further demonstrate this as a possible avenue, the authors should either demonstrate themselves or provide evidence from literature that Norvaline is safe.

L-Norvaline is being studied as a novel therapeutic agent for Alzheimer’s disease. Polis *et al*. (see Polis *et al*. *Neural Regen Res*. 2019. 4,9:1562-1572) demonstrated a reverse in cognitive decline in their mouse model of Alzheimer’s disease with 250mg/L L-norvaline in drinking water for 2.5 months (~2mM Norvaline). Subsequently, we used 5mM L-norvaline in our SCID mouse model of M. abscessus infection based on the mouse data and that 5mM = 10x MIC_90_ of norvaline against the editing deficient strain M. abscessus LeuRS^D436H^.

“Norvaline was dosed at 3.3 mg/kg (5 mM) which represents ~10X MIC_90_ *in vitro* against the editing deficient D436H mutant. Previously, norvaline had been shown to be an effective *in vivo* neuroprotective agent as an arginase inhibitor at 2 mM in a triple-transgenic mouse model of Alzheimer’s disease [41].”

17. Line 337 – The authors are trying to make the argument that using Norvaline in combination with EPT would be valuable, because rapid emergence of resistance caused EPT not to progress in phase 2 clinical study (UTI). However, this contradicts their previous findings, where they claim that resistance rates were lower than expected in M. abscessus. The UTI trial was probably on a different organism, therefore emergence of resistance may not be a problem for treatment of m. abscessus.

The rate of resistance to EPT in vitro in *M. abscessus* is much lower than what was discovered for *E. coli.* We agree with the reviewer that this high resistance in *E. coli* could be attributed to numerous factors like a different microorganism or a urinary tract infection rather than a lung infection. However, knowing that EPT failed a phase 2 trial, we believed that EPT would be overlooked had we not pursued an adjuvant to limit resistance.

18. Line345 – I do not think the enthalpy difference is strong enough to convincingly claim that EPT inhibits M. abscessus’ LeuRS more than Mtb’s. This needs to be assayed enzymatically.

The text has been modified to include other possible mechanisms to explain our phenotype which remains unexplained. We attempted to express and purify the full-length LeuRS however it was produced in the insoluble fraction in E. coli under various conditions assayed.

“Knowing that compounds with more enthalpically favourable binding are prioritized in hit-to-lead optimization [51], it may be possible that the discordance in enthalpy in part explains the difference in biological activity of EPT against the two organisms (MIC_90Mabs_ 0.063 µg/mL vs MIC_90Mtb_ 0.46 µg/mL). Other possibilities explaining the difference in whole-cell activity may be linked to differential expression level of *leuS* in both species, the lack of efflux pumps in *M. abscessus*, or the inability of *M. abscessus* to survive conditions that mimic leucine starvation, although these remain to be addressed.”

Reviewer #2: I thought this was a good manuscript covering an important area of drug discovery for this unmet medical need. A large number of experiments have been performed and the article tells a very complete story. I would support acceptance of this manuscript with a few minor revisions highlighted below

Thank you for the positive overall feedback

Reviewer #3: This well written manuscript describes a series of experiments that identified EPT as a potent inhibitor of Mycobacterium abscessus, confirmed the expected targeting of LeuRS, demonstrated therapeutic proof of concept in a mouse model of M. abscessus infection and tested the novel hypothesis that adjunctive norvaline could potentiate EPT efficacy and suppress emergence of EPT resistance by compromising survival of EPT-resistant LeuRS editing domain mutants. The authors conclude that the approach of combining oxaborole LeuRS inhibitors with leucine mimics hold promise for treatment of M. abscessus infections and potentially tuberculosis and other NTM infections.

The work is novel, comprehensive and potentially impactful for several difficult-to-treat mycobacterial infections. In addition to EPT, the oxaborole LeuRS inhibitor GSK3036656 has progressed to a phase 2 trial for tuberculosis. A few major and minor issues are described in the comments below.

Thank you for the positive overall feedback

**Part II – Major Issues: Key Experiments Required for Acceptance**
Please use this section to detail the key new experiments or modifications of existing experiments that should be absolutely required to validate study conclusions.

Generally, there should be no more than 3 such required experiments or major modifications for a "Major Revision" recommendation. If more than 3 experiments are necessary to validate the study conclusions, then you are encouraged to recommend "Reject".

Reviewer #1: (No Response)

Reviewer #2: None

Reviewer #3: The conclusion that the additive activity of norvaline and EPT observed in vivo is attributable to suppression of LeuRS mutants by the addition of norvaline is dubious. Given the extremely low frequency of spontaneous mutants observed in vitro, the limited growth of the bacteria in vivo and the limited bactericidal effect of EPT, these mutants are unlikely to have replaced the susceptible bacterial population in the lungs of mice after just 10 days of treatment. Thus, selective amplification of such mutants is unlikely to be the factor limiting the magnitude of the EPT effect in this model. This could be confirmed by testing the isolates obtained at the end of treatment for EPT resistance and LeuRS mutations. This comment is not intended to discount the observation of potentiation by norvaline but, rather, to challenge the proposed explanation. The proposed experiment could be avoided if the discussion is revised accordingly.

Text has been modified accordingly to include a secondary explanation for norvaline potentiation. The amino acid arginine is metabolized through the urea cycle by arginase or converted into nitric oxide by nitric oxide synthase (NOS), among other pathways. Thus, arginase and NOS compete for arginine. Chang and colleagues showed that when macrophages are given 5-20mM L-norvaline, a known arginase inhibitor (see Ming *et al*. 2009. *BMC Cardiovascular Disorders*. 9:12), NO levels increased. Increased NO production by macrophages might explain the lower bacterial burden observed in the SCID mouse model. (see Chang *et al*. 1998. *Am*. *J*. *Physiol*. 274: H342-H348.)

**Part III – Minor Issues: Editorial and Data Presentation Modifications**
Please use this section for editorial suggestions as well as relatively minor modifications of existing data that would enhance clarity.

Reviewer #1: (No Response)

Reviewer #2: Abstract, summary statement AND introduction should be reviewed to improve the flow of the text

The location of the resistant mutation (D436H) should be related to those seen in the clinical resistance in E.coli. In particular in relation to whether the same residues are seen and propose how the mutation causes resistance (in the crystallography section)

Text has been modified to include P. aeruginosa and E. coli LeuRS variants from two studies in relation to the variants identified in M. abscessus. See (Hernandez *et al*. *Antimicrob. Agents Chemother.* 2013. 57:3, 1394-1403.) and (O’Dwyer *et al*. *Antimicrob. Agents Chemother*. 2015. 59:1, 289-298.)

“This contrasts with LeuRS variants identified in *P. aeruginosa* such as T323P, T327P, and V429M from Hernandez *et al*. or LeuRS variants such as T322I, D326N, A414V, and R435H identified in a phase 2 clinical trial for complicated urinary tract infections caused by *E. coli* from O’Dwyer *et al*. [28,30]. However, the phase 2 clinical trial did identify *E. coli* isolates with a LeuRS D436A variant. (*M. abscessus* numbering).”

“In addition, R435 (R449 in *M. tuberculosis*) hydrogen bonds with the ethoxy oxygen of EPT and packed with the ribose of AMP (Fig 4a). The packing interaction on the ribose of AMP is underscored from *E. coli* mutants with R435H variants that lead to resistance early in the phase 2 clinical trial for complicated urinary tract infections.”

*“M. tuberculosis* LeuRS T337 is modelled in the latter position, but inspection of deposited maps also indicates multiple rotomeric conformations. Variants at T322, T323 and T327 were identified in *P. aeruginosa* and *E. coli* mutants resistant to benzoxaboroles, which supports the importance of the threonine rich region for editing activity [32].”

Is reference 40 the correct reference for 2mM in vivo treatment of norvaline, if it is then it needs to be made clear this its activity is not for mycobacterial infection models.

Reference is correct. Text has been modified to state the activity is not for mycobacterial infection but rather in vivo activity against arginase in a triple-transgenic mouse model of Alzheimer’s disease.

“Previously, norvaline had been shown to be an effective *in vivo* neuroprotective agent as an arginase inhibitor at 2 mM in a triple-transgenic mouse model of Alzheimer’s disease [41].”

An explanation needs to proposed for why in vivo synergy is seen, I am not an expert in this area but in a study this short it seems unlikely to me that when there is no effect on MIC, synergy would be related to effects on escape resistance when only infecting with 106 bacteria and frequency of resistance is only 2 x-9

Thank you to the reviewer for this comment. This has been addressed in the answer to a previous reviewer.

Text will be modified accordingly to include a secondary explanation for norvaline potentiation. The amino acid arginine is metabolized through the urea cycle by arginase or converted into nitric oxide by nitric oxide synthase (NOS), among other pathways. Thus, arginase and NOS compete for arginine. Chang and colleagues showed that when macrophages are given 5-20mM L-norvaline, a known arginase inhibitor (see Ming *et al*. 2009. *BMC Cardiovascular Disorders*. 9:12), NO levels increased. Increased NO production by macrophages might explain the lower bacterial burden observed in the SCID mouse model. (see Chang *et al*. 1998. *Am*. *J*. *Physiol*. 274: H342-H348.)

There are two publications this year relating to M abscessus and epetraborole. While this manuscript expands on these studies by looking at norvaline it should mention these earlier publications

Both publications have been added. See (Ganapathy *et al*. *Antimicrob Agents Chemother*. 2021) and (Kim *et al.* *Int J Mol Sci*. 2021. 22, 11)

“Our data and two recent publications [42,43] confirmed the activity of benzoxaboroles against mycobacteria using zebrafish and murine models of infection and we showed that benzoxaboroles can be potentiated when combined with norvaline.”

Reviewer #3: The prospects for incorporating norvaline into clinical use for the adjunctive treatment of mycobacterial treatment could be discussed in more detail. For example, what is the relevance of the 5 mM concentration/dose tested in vitro and in vivo in light of what is known of the human exposures that may be safely attained and tolerated?

Thank you to the reviewer for highlighting the safety of norvaline. This has been addressed in the answer to a previous reviewer.

L-Norvaline is being studied as a novel therapeutic agent for Alzheimer’s disease. Polis *et al*. (see Polis *et al*. *Neural Regen Res*. 2019. 4,9:1562-1572) demonstrated a reverse in cognitive decline in their mouse model of Alzheimer’s disease with 250mg/L L-norvaline in drinking water for 2.5 months (~2mM Norvaline). Subsequently, we used 5mM L-norvaline in our SCID mouse model of M. abscessus infection based on their mouse model for Alzheimer’s disease and that 5mM = 10x MIC_90_ of norvaline against the editing deficient strain M. abscessus LeuRS^D436H^.

“Norvaline was dosed at 3.3 mg/kg (5 mM) which represents ~10X MIC_90_ *in vitro* against the editing deficient D436H mutant. Previously, norvaline had been shown to be an effective *in vivo* neuroprotective agent as an arginase inhibitor at 2 mM in a triple-transgenic mouse model of Alzheimer’s disease [41].”

EPT concentrations are alternately described in uM or ug/ml in different sections of the manuscript; one unit should be adopted throughout, with the translation of uM to ug/ml encouraged if uM is used

All concentrations have been converted to ug/mL.

Lines 157-70: in this paragraph, figs referenced should be figs 2a-d rather than figs 1a-d

Figure references have been changed accordingly.

Line 322: suggest revision to “~10X MIC90 in vitro against the D436H mutant” or something similar to clarify this MIC is against the mutant

Text has been modified to clarify dose of norvaline is based on the LeuRS editing mutant D436H.

Norvaline was dosed at 3.3 mg/kg (5 mM) which represents ~10X MIC_90_ *in vitro* against the editing deficient D436H mutant.

Line 323: suggest revision to “effective in vivo as a neuroprotective agent” or something similar to clarify that this statement of efficacy applies to a different, non-infectious disease model

Text has been modified to provide clarity on the effect norvaline has on the mouse model of Alzheimer’s disease.

“Norvaline was dosed at 3.3 mg/kg (5 mM) which represents ~10X MIC_90_ *in vitro* against the editing deficient D436H mutant. Previously, norvaline had been shown to be an effective *in vivo* neuroprotective agent as an arginase inhibitor at 2 mM in a triple-transgenic mouse model of Alzheimer’s disease [41].”

Line 386: please indicate which strain was engineered for luminescence

Text has been modified to include the ATCC strain of M. abscessus and the plasmid used for lux.

“MMV Pathogen Box, MMV Pandemic Response Box, and GSK TB-active libraries were screened against *M. abscessus* ATCC 19977 strain which constitutively expressed the *luxCDABE* operon (*M. abscessus* lux) at 10 µM in duplicate in 96-well flat-bottom plates in 7H9 complete media.”

Lines 752-4: duplicate of ref 30

Duplicate ref has been removed

Lines 811-3: duplicate of ref 26

Duplicate ref has been removed

Fig 1 legend: for fig 1c, change “upper” and “lower” to “left” and “right”

Figure 1 legend has been modified with “left” and “right”.
